# Novel Method of Semantic Segmentation Applicable to Augmented Reality

**DOI:** 10.3390/s20061737

**Published:** 2020-03-20

**Authors:** Tae-young Ko, Seung-ho Lee

**Affiliations:** 1 Department of Electronic Engineering, Hanbat National University, Daejeon 34158, Korea; 2 Department of Electronics & Control Engineering, Hanbat National University, Daejeon 34158, Korea

**Keywords:** semantic segmentation, modified dilated residual network, atrous pyramid pooling module, backpropagation, augmented reality, convolutional neural network, fully convolutional network

## Abstract

This paper proposes a novel method of semantic segmentation, consisting of modified dilated residual network, atrous pyramid pooling module, and backpropagation, that is applicable to augmented reality (AR). In the proposed method, the modified dilated residual network extracts a feature map from the original images and maintains spatial information. The atrous pyramid pooling module places convolutions in parallel and layers feature maps in a pyramid shape to extract objects occupying small areas in the image; these are converted into one channel using a 1 × 1 convolution. Backpropagation compares the semantic segmentation obtained through convolution from the final feature map with the ground truth provided by a database. Losses can be reduced by applying backpropagation to the modified dilated residual network to change the weighting. The proposed method was compared with other methods on the Cityscapes and PASCAL VOC 2012 databases. The proposed method achieved accuracies of 82.8 and 89.8 mean intersection over union (mIOU) and frame rates of 61 and 64.3 frames per second (fps) for the Cityscapes and PASCAL VOC 2012 databases, respectively. These results prove the applicability of the proposed method for implementing natural AR applications at actual speeds because the frame rate is greater than 60 fps.

## 1. Introduction

The Fourth Industrial Revolution is accelerating the research and development of artificial intelligence and robots that think like humans. Consequently, there is growing interest in research on the movement, judgment, and operation of virtual reality (VR) and augmented reality (AR), autonomous driving, medical robots, and drones [1]. The research in these fields is based on the analysis of images captured by cameras that assume the role of the human eye. The primary task for image analysis is semantic segmentation, in which labeling is performed to determine the class to which each pixel belongs [2].

Semantic segmentation is a technique for dividing images into pixels according to pre-learned classes. It is not merely about categorizing images into classes, but is also a high-level technique for understanding in its entirety the scenes in images, and is one of the core computer vision technologies required to understand the visual environment fully [3]. A semantic segmentation algorithm requires efficient speed for quick interaction or response and high accuracy for accurate judgment. For example, speedy semantic segmentation and accurate judgment are essential for safe control driving decisions and collision avoidance in autonomous driving. However, it is difficult to perform accurate semantic segmentation in real time with photographed images [4]. To solve these problems, a robust algorithm that is flexible to changes in appearance is needed. At the same time, various situational data must be considered to distinguish objects from complex backgrounds.

Furthermore, segmentation is applied as an essential element in fields that require real-time image semantic segmentation such as VR, AR, autonomous driving, medical robots, and drones. In particular, autonomous driving requires segmentation recognition speeds of 100 frames per second (fps) or greater. However, AR applications require a segmentation recognition speed of only approximately 60 fps, which is appropriate for the walking speed of humans. Therefore, the semantic segmentation method proposed in this study is a suitable solution for AR.

The main contributions of this study are as follows. (1) A diluted residual network process that extracts feature point maps from the original image using atrous convolution, which transforms the composition to maximize spatial information and extracts feature points. This improves the accuracy of semantic segmentation by reducing the loss of space information. (2) An atrous pyramid pooling module process that applies various atrous convolutions to feature point maps to effectively extract objects with small areas of the image. In addition, to reduce the time required, the atrous convolution is placed in parallel to extract the feature points and is then stacked in the shape of a pyramid. During this process, small feature point maps are stacked equally with up-sampling. When the various feature point maps are stacked in the pyramid shape, 1 × 1 convolution is applied to make them into one-channel feature point maps. This improves accuracy because even small objects can be extracted more accurately. (3) A diluted residual network backpropagation process that compares the semantic segmentation obtained from the final feature point map with the resulting image provided by the database and, if a certain error rate occurs, applies it to the convolution performed in the second process to reduce the error rate, ultimately enhancing the accuracy of the semantic segmentation.

## 2. Literature Review

Among the early studies on the accuracy of semantic segmentation, histograms of oriented gradient (HOG) [5] and scale-invariant feature transform (SIFT) [6] performed semantic segmentation through the recognition of handcrafted features using a classifier such as support vector machine (SVM) [7]. Subsequently, instead of handcrafted features and classifiers, studies have focused on deep convolutional neural networks (CNNs) that extract and classify data-driven features using large-scale learning. These studies of data-based classification achieved higher performance than existing studies in various computer vision fields. Recently, however, studies of semantic segmentation using deep learning have begun receiving attention. Deep learning-based methods that show excellent results in image recognition and classification and semantic segmentation are being developed. Image classification methods perform classification on entire images, whereas image segmentation methods perform segmentation in small pixel units and therefore require considerable memory and high accuracy, resulting in high computational complexity. To address this problem, four representative network structures are used in semantic segmentation [8]. The first involves learning using a pyramid structure by changing input images in various scales. The second obtains results by up-sampling through pooling and decoding using the step-by-step encoding of feature maps. The third is a network structure that changes the filtering kernel itself to achieve high accuracy, even with diverse sizes. The last is a structure that combines information to obtain new features after pooling feature maps in various resolutions.

Meanwhile, in consideration of the need for real-time semantic segmentation algorithms [4,9,10,11], three main approaches have been applied to improve the speed and accuracy of semantic segmentation algorithms. The first approach, presented in the paper “ICNet for Real-Time Semantic Segmentation on High-Resolution Images” by Wu et al. in 2018, reduces computation complexity by cutting or resizing input images in a limited scale [12]. This method is simple and effective, but the accuracy, in terms of indicators and visualization, decreases near boundaries. The second approach rearranges the channels of the network to increase the inference speed in the network, instead of adjusting the scale of the input images [9,10,13]. However, this diminishes the spatial capacity of the algorithm. Finally, efficient neural network (ENet) [10] increases the speed of the algorithm in the final stage of the network, but this approach has the disadvantage of lowering the accuracy of semantic segmentation because the down-sampling work in the last step is renounced. 

All of the above approaches reduce accuracy while increasing speed. Accuracy in a semantic segmentation algorithm requires assigning the accurate category label to each pixel and the difficulty in achieving accuracy is closely related to the diversity of images and labels. The current semantic segmentation methods are based on the fully convolutional network (FCN) because it has no limit on the size of the input images and more spatial information can be kept [14]. Below, we examine the representative semantic segmentation methods that are currently being used.

The new paradigm of FCN with the decoder (CNN)–decoder structure that differs from that of deep neural networks [15,16,17,18] has appeared in the semantic segmentation field. The first paper on FCN was published by Long et al. under the title “Fully Convolutional Networks for Semantic Segmentation” [14]. It has since been cited by numerous studies because the FCN showed excellent performance in semantic segmentation without using any difficult techniques. The representative networks (VGGNet, AlexNet [15], GoogleNet) of existing classification methods have problems where the fully connected layer at the end can only receive inputs of a certain size and the location information disappears when it passes through the fully connected layer. The FCN was developed based on the idea that the fully connected layer can be replaced with a 1 x 1 convolution, with which the location information can be retained. In an FCN, the size of the input images is not limited because every network is a convolution network. Furthermore, because entire images are processed at once rather than in patch units, the required time is shortened by the effect of reduced computation. Thus, the method is still widely used. However, one disadvantage of the FCN combined with 1 × 1 convolution, in the end, is that, because images are reduced through pooling, it is difficult to preserve detailed location information because the values of the feature map correspond to the many pixel values of the result images. To overcome this problem, networks with a U-shape structure, in which the information is applied before the image reduction is applied to the result have been published [9,14,19,20,21]. Among them, Ronneberger et al. published “U-net: Convolutional Networks for biomedical image segmentation” in 2015 [21]. The structure of U-net has added skip connection, gradual up/down-sampling, and other features. 

In addition to U-net, there are many methods that use changed U-shapes. Some of them create U-shape networks using the deconvolution layer [9,19]. The global convolution network [22] combines the U-shape structure with a “large kernel.” LRR [20] uses the Laplacian pyramid reconstruction network. To improve performance, refine net [19] adds an improved multi-path structure. The U-shape structure has the advantage of maintaining the spatial information better than the FCN, but it still has limitations in restoring the lost spatial information. The biggest problem of FCN and U-net is that the size of the feature map is reduced, and much location information is lost. As this problem is caused by network pooling, algorithms to replace pooling have been studied [23,24,25,26]. Among them, Yu et al. published “Multi-Scale Context Aggregation by Dilated Convolutions” in 2015 [23]. Dilated convolution originates from the atrous algorithm, which is used in the wavelet decomposition algorithm, and is also called atrous convolution. Dilated convolution has the advantages of increasing the size of the receptive field with no loss in resolution and it controls the amount of computation by filling all parts, except the red points, with zero.

Furthermore, the size of the feature map extracted by using dilated convolution is four times larger than that obtained when using general convolution [26]. However, one disadvantage is that every step must be tested through experiments in the process of deciding on a threat. To address the problem that the loss of location information seen whenever pooling is performed is different for each filter size (even when dilated convolution is used); methods to extract information for each filter and then combine them later have been studied. The spatial pyramid pooling network (SPPNet) was presented by He in 2015. SPPNet uses the bag of words (BoW) [27] concept, in which objects can be distinguished better when many small features are used, instead of depending on thick and strong features for classifying specific objects. As with BoW, SPPNet uses small feature maps derived from multiple steps of pyramids as input for the fully connected layer. The final convolutional layer of an existing neural network such as ZFNet [28] is converted into a pyramid pooling layer, and in the final pyramid layer, the results of the last convolutional layer are divided into multiple steps of pyramids. 

Chen et al. proposed the atrous spatial pyramid pooling (ASPP) module that collects situation information from various regions in the images [25]. PSPNet utilizes the pyramid scene pooling (PSP) module, which includes various standards of the average pooling layer [29]. DeepLabv3 uses the ASPP module with global average pooling to extract the situation information of images [26]. According to a paper published by Zhang et al. in 2017, the adaptive image situation information is obtained by improving the neural network using scale adaptive convolution [30]. The discriminative feature network (DFN) encodes the situation information of images by adding global pooling to the U-shape structure [31]. Chen et al. announced DeepLabv3+, which combines encoder/decoder, dilated convolution (atrous convolution) and spatial pyramid pooling, which were used in the research on semantic segmentation [32]. DeepLabv3+ can arbitrarily control the resolution of the feature map extracted from the encoder, which is impossible in the general encoder–decoder structure, using atrous convolution. Furthermore, it applies depth-wise separable convolution to the ASPP module and decoder.

There are many reasons for using the dilated convolution and atrous pyramid pooling module as methods for accurate semantic segmentation in this study. First, the accuracy of semantic segmentation can be improved by reducing the loss of spatial information. Second, small objects can be extracted more accurately through convolutions of various rates. Thus, we used dilated convolution and the atrous pyramid pooling module to achieve semantic segmentation accurately. In addition, we further improved the accuracy of semantic segmentation by introducing backpropagation.

## 3. Novel Method of Semantic Segmentation Applicable to Augmented Reality (AR)

**Figure 1:**
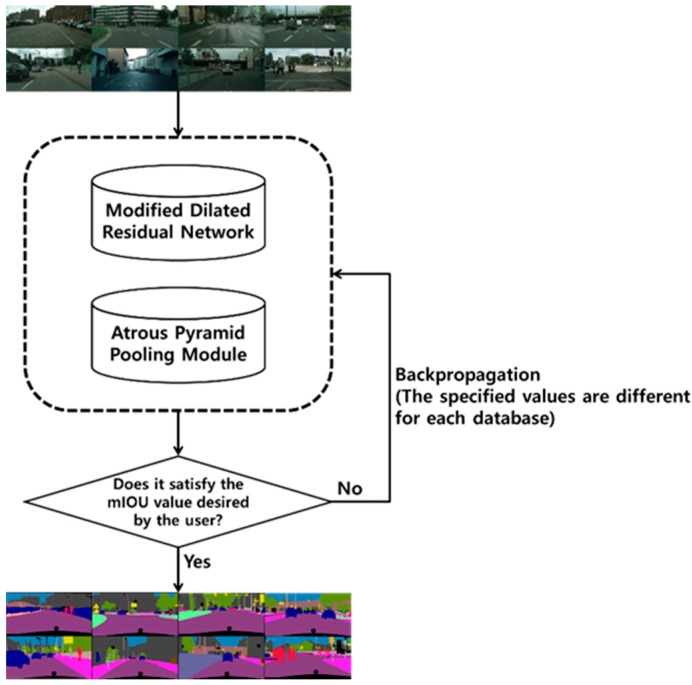
Overall structure of the proposed semantic segmentation method.

In this study, we used a modified dilated residual network, atrous pyramid pooling module, and backpropagation to improve the accuracy of semantic segmentation. Specifically, we first applied dilated convolution and the atrous pyramid pooling module to improve accuracy by extracting feature maps that retain considerable spatial information. Second, we improved accuracy by repeatedly performing backpropagation with an accuracy value in terms of mean intersection over union (mIOU) desired by the user. Figure 1 shows the overall structure of the proposed novel method of semantic segmentation applicable to AR.

### 3.1. Acquiring Semantic Segmentation Image

**Figure 2:**
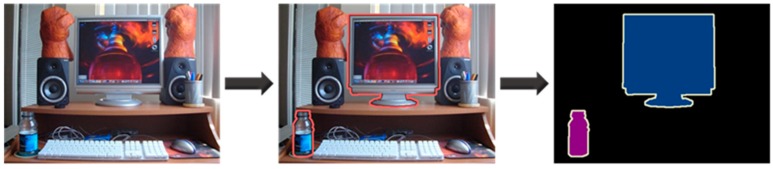
General semantic segmentation image acquisition process.

To acquire the semantic segmentation images, the objects must be manually classified and labeled using general images obtained with a camera (as shown in Figure 2). However, we used two standard databases for objective evaluation of the semantic segmentation method.

**Figure 3:**
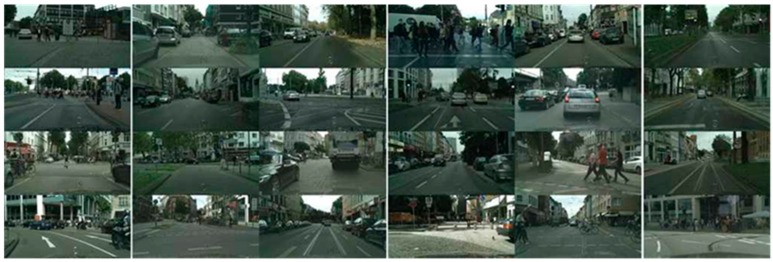
Cityscape database image.

The Cityscapes [33] database is composed of images labeled as objects for complex scenes in many different cities, as shown in Figure 3. In this study, we acquired images provided by the Cityscape database to evaluate the semantic segmentation method objectively.

**Figure 4:**
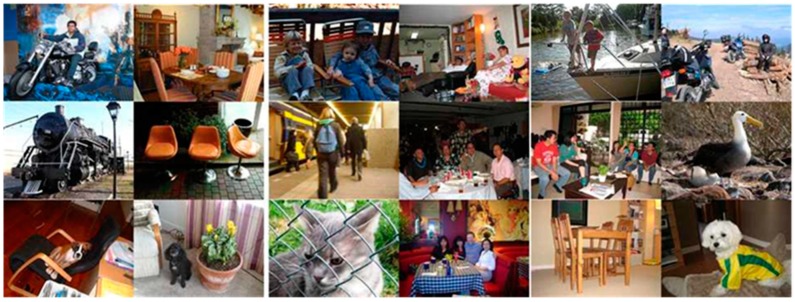
PASCAL VOC 2012 database images.

The PASCAL VOC 2012 [34] database is composed of 20 classes in total; Figure 4 shows images from each class. In this study, we acquired the images provided by the PASCAL VOC 2012 database to evaluate the semantic segmentation method objectively. The sizes of all semantic segmentation images used in this experiment were adjusted to 513 × 513 for consistency.

### 3.2. Modified Dilated Residual Network

**Figure 5:**
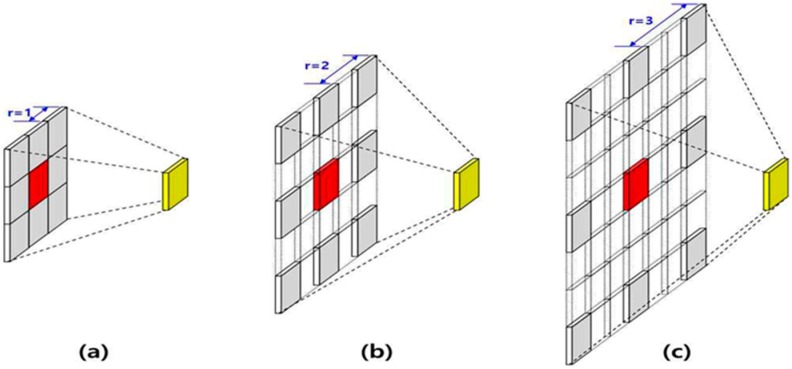
(**a**) Atrous convolution when r = 1, (**b**) Atrous convolution when r = 2, (**c**) Atrous convolution when r = 3.

Convolution is the most representative algorithm for extracting features from images while maintaining as much spatial information as possible. One of the methods, atrous convolution, was named from the French word “atrous” (having a hole). Influenced by wavelet analysis, zero-padding was added in the filter to increase the window size without increasing the number of weights. Atrous convolution captures large features with the same amount of computation as that used in general convolution and can extract more spatial features by using atrous convolutions with various expansion ratios in parallel. Equation (1) describes the case in which the rate is one, representing general convolution, and Equation (2) describes the case in which the rate is larger than one, representing atrous convolution. Figure 5 illustrates the atrous convolutions in which the rate is one, two, or three. In Equations (1) and (2), F is a discrete function, k is a discrete filter of size ${\left(2r+1\right)}^{2}$, and l is a dilation factor [23].

(1) $$\left(F*k\right)\left(p\right)=\underset{s+t=p}{\sum }F\left(s\right)k\left(t\right).$$

(2) (F*kl)(p)=∑s+lt=pF(s)k(t).

**Figure 6:**
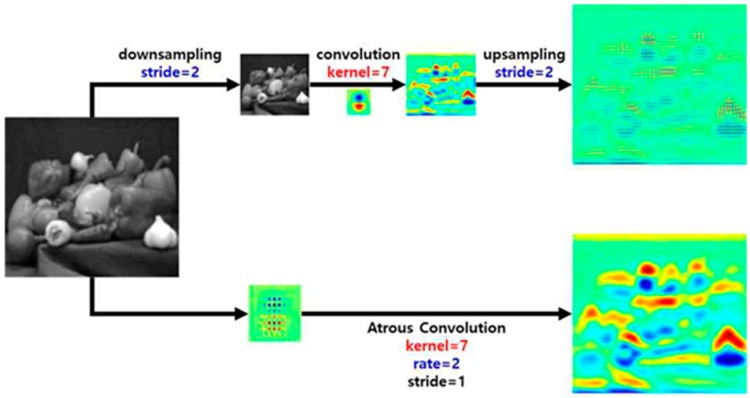
Comparison of semantic segmentation between general convolution and atrous convolution.

The accuracy decreases if semantic segmentation is performed with small feature maps obtained using a general convolution network. Figure 6 shows the difference between performing semantic segmentation through down-sampling, convolution, and up-sampling and performing semantic segmentation through atrous convolution. The illustration of general convolution shows that the resolution of semantic segmentation is decreased by up-sampling, with loss of spatial information. However, atrous convolution can minimize the loss of spatial information and increase the resolution by performing convolution with a large receptive field.

**Figure 7:**
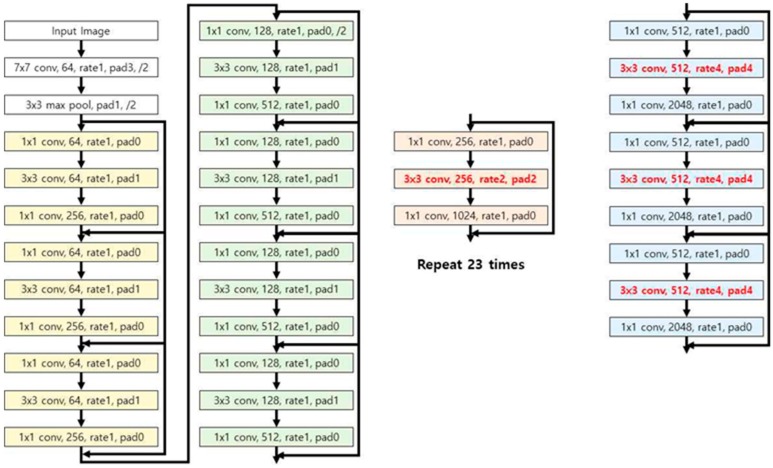
Structure of the modified dilated residual network applied in this study.

In this study, feature maps were extracted using a modified dilated residual network constructed by modifying the ResNet-101-step network structure to improve accuracy. The modified dilated residual network learns long-distance features without depending on the pooling function by expanding the kernel with empty weights, and maintains more detailed elements of a higher space frequency even without pooling. Figure 7 shows the structure of the modified dilated residual network applied in this study. The network was built by converting the convolution of Group 4 and Group 5 of the Resnet-101 steps to dilated convolution with two and four rates. Through this, feature maps with better spatial information can be extracted.

### 3.3. Atrous Pyramid Pooling Module

R-CNN [35], a representative segmentation method, generates several thousand extract region proposals with a selective search for images and then performs segmentation through CNN. However, R-CNN has the disadvantage that it takes a long time because CNN must be applied to each of several thousand extract region proposals. To address this problem, SPP was applied to the feature maps extracted from the last layer of convolution instead of from the pooling layer. The SPP module extracts variously sized features through convolution and global max pooling by applying various strides for the feature maps obtained through convolution. In this process, vectors of the same size are output even if images of various sizes are input, if the segmentation size is the same. Subsequently, various feature maps are combined into a pyramid and the resulting image is obtained again through convolution. In 2017, the pyramid scene parsing network (PSPNet) was published at the Computer Vision and Pattern Recognition (CVPR) Conference. In PSPNet, the PSP obtains four images of dimensions 1 × 1, 2 × 2, 3 × 3, and 6 × 6 through image pooling from feature maps, and performs segmentation of various objects by stacking feature maps of a wide range in a pyramid shape through convolution.

**Figure 8:**
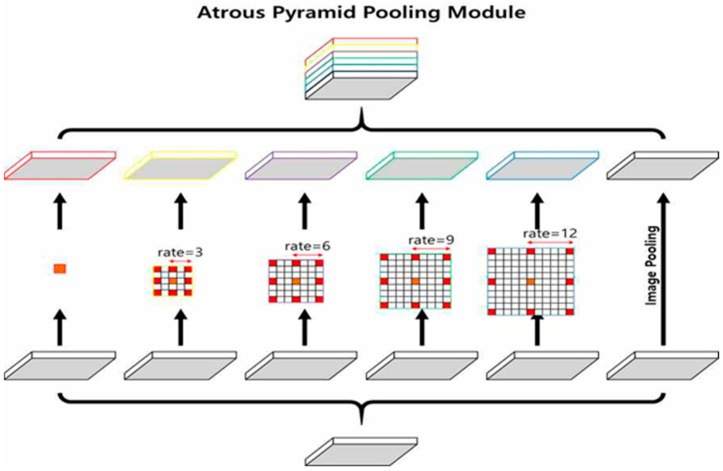
Structure of the atrous pyramid pooling module applied in this study.

The modified dilated residual network described in Section 3.1 creates feature maps by only extracting important features, while preserving the features of the space domain from the entire input images. However, to increase accuracy in semantic segmentation, it is critical to extract even small objects accurately. In the current semantic segmentation field, segmentation is very difficult when small objects are arranged in a complicated fashion. Therefore, to solve this problem, we used the atrous pyramid pooling module, which was transformed from the pyramid sense pooling of the PSPNet. The atrous pyramid pooling module, illustrated in Figure 8, has the following characteristics. Feature maps are extracted by applying five types of atrous convolutions to the 28 × 28 feature maps obtained through the modified dilated residual network in parallel. The atrous convolutions applied here consist of general convolution with rate = 1, atrous convolutions with rate = 3, rate = 6, and rate = 9, and image pooling applied to the extracted feature maps. Subsequently, a pyramid is stacked with these five feature maps, and 1-channel feature maps are extracted by applying a 1 × 1 convolution to extract even small objects accurately.

In this study, we employed the atrous pyramid pooling module that maintains the spatial information of various sizes by applying convolutions of various rates, and we observed improved accuracy.

### 3.4. Backpropagation

**Figure 9:**
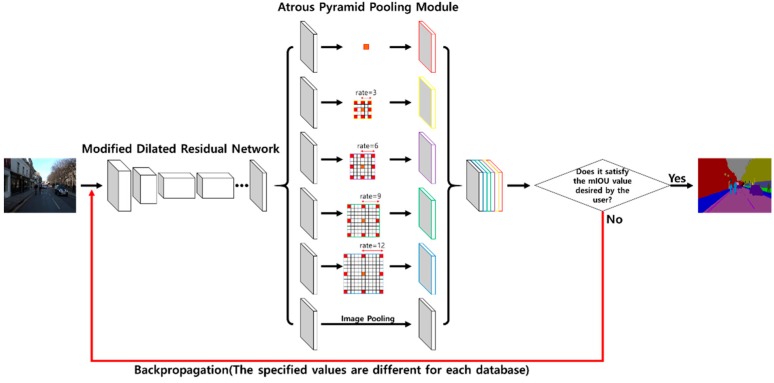
Structure of the backpropagation applied in this study.

In our method, if the loss rises above a certain amount, it is reduced through backpropagation. Backpropagation is performed by comparing the input image and the result of applying the modified dilated residual network, but the result of the atrous pyramid pooling module may affect the accuracy. Therefore, backpropagation is performed (Figure 9) by using the loss obtained from comparing the result of the atrous pyramid pooling module with the ground truth provided by the database.

**Figure 10:**
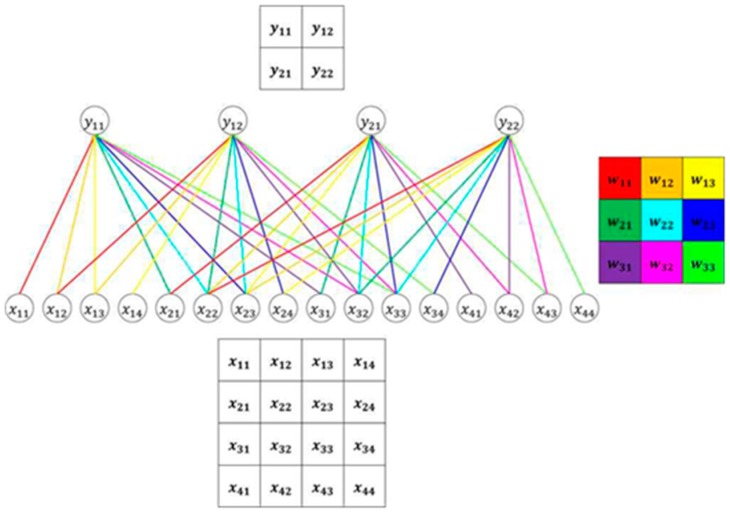
Example of convolution.

The backpropagation process is as follows. The CNN extracts features while the filter slides the input data, compresses them by max pooling or average pooling, and sends them to the next layer. The general structure of the CNN causes the process to repeat. The input is a 5 × 5 matrix, in which $x_{\mathrm{ij}}$ denotes the element in the $i_{\mathrm{th}}$ row and $j_{\mathrm{th}}$ column. When convolution is performed on this input with a filter size of 3 × 3, the output has the size 2 × 2. Figure 10 illustrates an example in which 3 × 3 convolution is performed when the input image is 5 × 5, indicating that the value $y_{11}$ is output by the convolution of $x_{11}$, $x_{12}$, $x_{13}$, $x_{21}$, $x_{22}$, $x_{23}$, $x_{31}$, $x_{32}$, and $x_{33}$.

**Figure 11:**
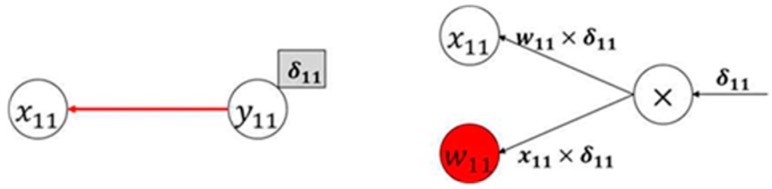
Karpathy calculation graph of $x_{11}$ backpropagation.

Figure 11 shows backpropagation based on the convolution structure. In the forward process, $x_{11}$ performs convolution only with weight $w_{11}$ of the 3 × 3 filter; backpropagation is only performed once. This backpropagation process can be represented in a Karpathy calculation graph, as shown in Figure 11. The gradient of $x_{11}$ can be determined by multiplying the inflow gradient $\delta_{11}$ by the local gradient ($w_{11}$), which indicates the change in the other party. Likewise, the gradient of $w_{11}$ can be determined by multiplying the inflow gradient $\delta_{11}$ by the local gradient ($x_{11}$).

**Figure 12:**
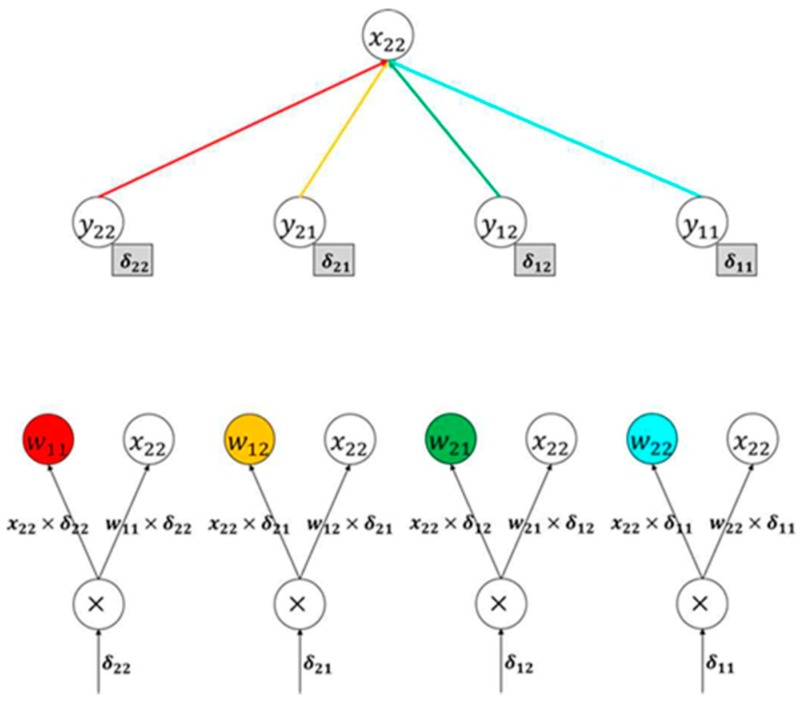
Karpathy calculation graph of $x_{22}$ backpropagation.

When we examined $x_{22}$ using the same method, it can be seen that the amount of computation increased compared to $x_{11}$, but the calculation process was the same. Figure 12 shows the backpropagation Karpathy calculation graph of $x_{22}$.

**Figure 13:**
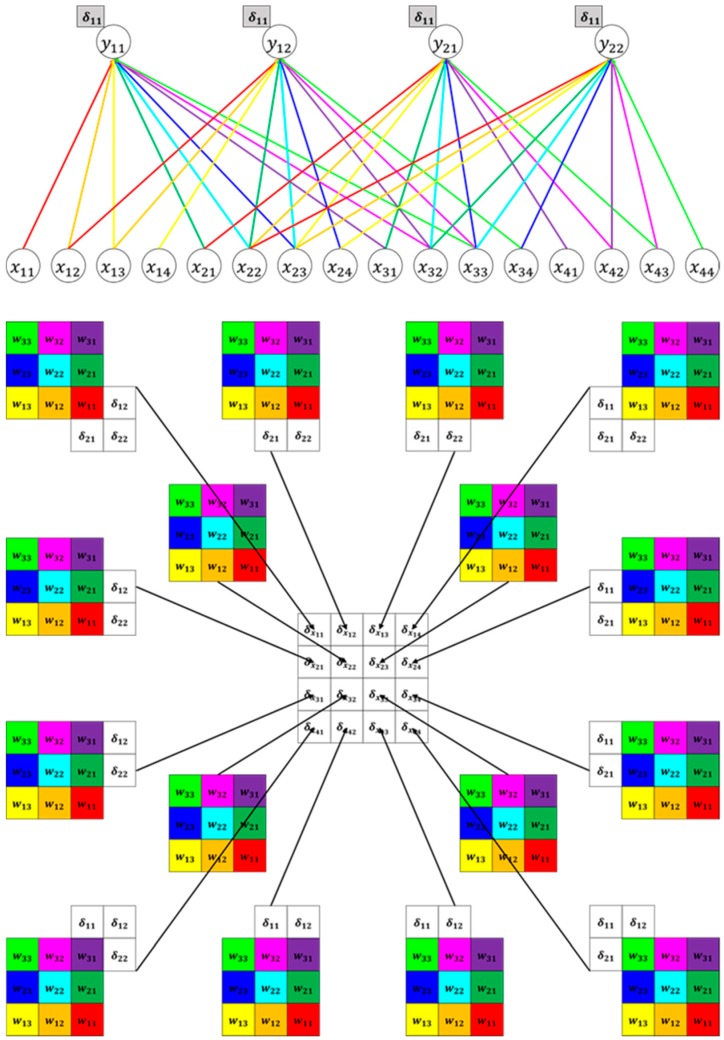
Backpropagation image.

Figure 13 shows a simpler method of calculating the gradient, because it is difficult to substitute the backpropagation method every time. The gradient of the input vector can be obtained by performing convolution of the gradient matrix by inverting the elements of the filter used when creating the convolution layer. For example, the gradient of $x_{11}$ can be determined using Equation (3), and the operation marked by a red square in Figure 13 can be expressed, as in Equation (3).

(3) $$x_{11}=w_{11}\times \delta_{11}.$$

For the gradient of the filter, the first element of the inflow gradient matrix, $\delta_{11}$, is connected with $x_{11}$, $x_{12}$, $x_{21}$, and $x_{22}$. Hence, the gradient of the filter can be determined by multiplying the inflow gradient ($\delta_{11}$, $\delta_{12}$, $\delta_{21}$, $\delta_{22}$) by the local gradient. Equation (4) is the equation used to obtain the slope $\delta_{\omega_{11}}$ of weight $\omega_{11}$.

(4) $$x_{w_{11}}=x_{11}\delta_{11}+x_{12}\delta_{12}+x_{21}\delta_{21}+x_{22}\delta_{22}.$$

**Figure 14:**
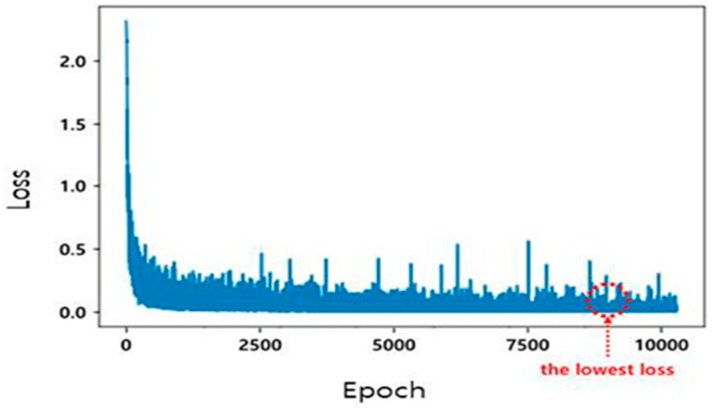
Loss value according to epoch for the Cityscapes database.

Figure 14 shows the loss that results in the repetitive learning process for the Cityscapes database. A total of 1525 datasets were learned, and the smallest loss in 10,000 epochs was selected.

**Figure 15:**
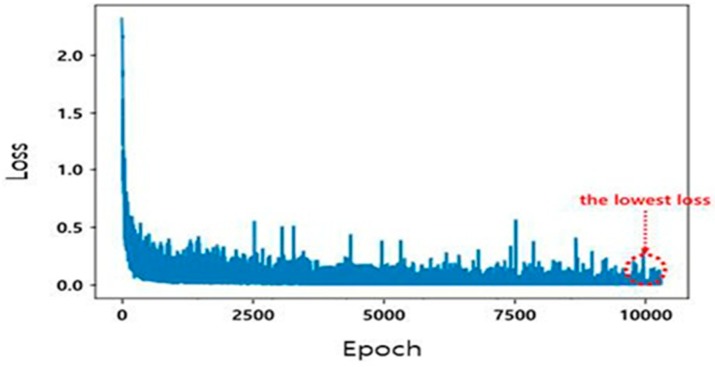
Loss value according to epoch for the PASCAL VOC 2012 database.

Figure 15 shows the loss that resulted in the repetitive learning process for the PASCAL VOC 2012 database. A total of 4318 datasets were learned, and the smallest loss in 10,000 epochs was selected.

## 4. Result and Discussion

In this study, we evaluated the time required for the proposed semantic segmentation method, according to the number of segmentations and the crossing rate (accuracy) between the predicted boundary box and the ground truth boundary box. In the learning process, the Cityscapes database and the PASCAL VOC 2012 database were learned by applying the modified dilated residual network and the atrous pyramid pooling module. In the performance process, semantic segmentation was performed based on the learned data.

The Cityscapes database and the PASCAL VOC 2012 database were used to evaluate the objective performance of the proposed semantic segmentation method. The hardware used in this experiment was an Intel(R)Core(TM) i7-9700K 3.60 GHz CPU, 16 GB RAM, and NVIDIA GeForce RTX2080 Ti(V-RAM11GB) GPU. We used JetBrains PyCharm Community Edition 2019.2.4 as the development tool on the Windows 10 Pro 64-bit operating system. In addition, we used the TensorFlow 1.13.1, CUDA8.0, and cuDNN 7.6.4 libraries.

### 4.1. Cityscapes Database Result

The Cityscapes database is an open standard database of urban street images that has been used as a benchmark for comparisons in prior studies. It comprises 5000 images in more than 30 classes, taken on different days and times in more than 50 cities. In this study, we performed semantic segmentation experiments with all the images in this database.

To evaluate the accuracy and required time, the images of the Cityscapes database were adjusted for this experiment to a size of 513 × 513. Furthermore, to evaluate the objective reliability of the proposed semantic segmentation method, it was compared with the results in “Encoder–Decoder with Atrous Separable Convolution for Semantic Image Segmentation,” published by Chen et al. in 2018 [32].

Table 1 shows the accuracy results for the semantic segmentation method proposed in this paper and the methods published in other papers in the same environment, based on Chen et al. [32]. As shown in the table, the proposed semantic segmentation method exhibited higher accuracy than the other methods. It appears that the DeepLabv3+ proposed by Chen et al. [32] could not perform semantic segmentation more accurately because it only considers the forward direction and does not perform backpropagation. The semantic segmentation method proposed in this paper exhibited higher accuracy than other methods because it performed backpropagation when the mIOU of the final image was not greater than a certain value.

**Figure 16:**
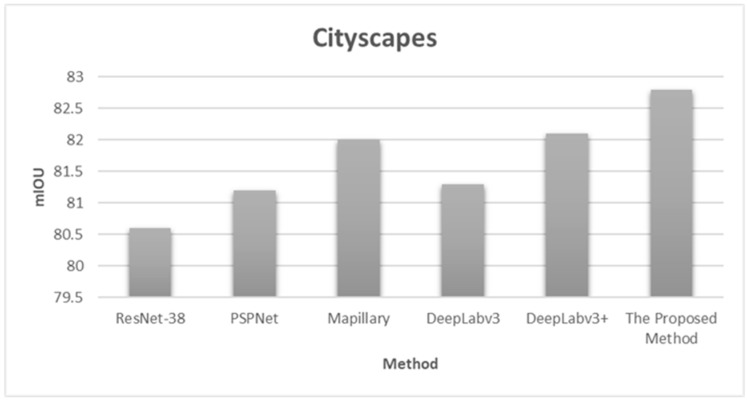
Comparison of the accuracy (mIOU) of the proposed method and methods from the literature on images in the Cityscapes database.

**Figure 17:**
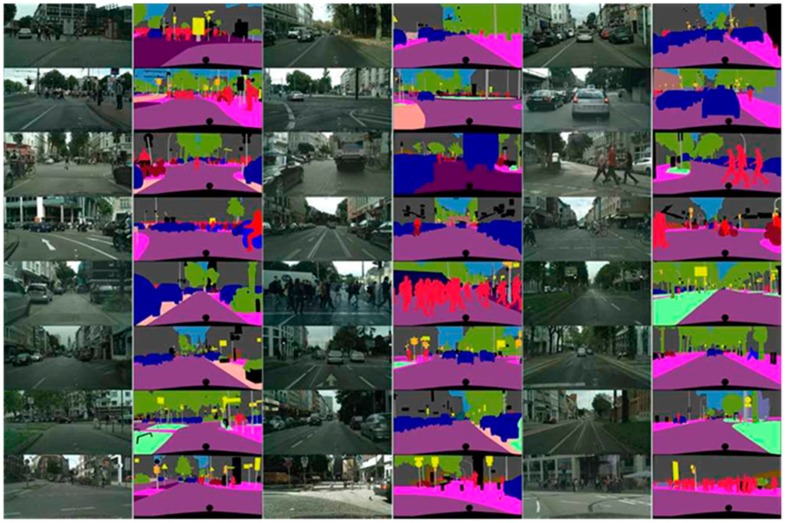
Results from the proposed method for the Cityscapes database photos.

Figure 16 graphically compares the accuracy (mIOU) for the proposed method and other algorithms that were evaluated in the same environment by Chen et al. [32]. The figure confirms that the semantic segmentation method proposed in this paper had a higher accuracy than the other semantic segmentation methods. Figure 17 shows the experimental results of the semantic segmentation method proposed in this paper for the Cityscapes database.

In addition, we measured the required time for the proposed semantic segmentation method with the Cityscapes database. However, we did not compare this result with those of other methods because the required time can vary with the performance of the hardware on which the program is executed. The time it takes to perform semantic segmentation with the images of the Cityscapes database as input was also measured. Table 2 outlines the frame rate determined by the number of images for which semantic segmentation was performed by the proposed method with the Cityscapes database, and it was 61 fps. As the frame rate exceeded 60 fps, it is applicable to the AR field for following human motion.

### 4.2. PASCAL VOC 2012 Database Result

The PASCAL VOC 2012 database was used for the PASCAL VOC Challenge. The database consists of 20 classes in total because the segmentation databases have been increased, or more detailed comments have been added. In this study, 4318 images with segmentation comments provided by the database were used. To evaluate accuracy and time, the images used in the experiments were adjusted to a size of 513 × 513. The objective reliability of the proposed semantic segmentation method was compared with that of Chen et al. [32].

Table 3 lists the accuracy results for the methods evaluated in Chen et al. [32] and the method of semantic segmentation proposed in this paper. As shown in the table, the accuracy result for the proposed semantic segmentation method was higher than those seen with other methods. As with the Cityscapes case, it appears that DeepLabv3+, as proposed by Chen et al. [32], could not perform semantic segmentation more accurately because it only considers forward direction and does not perform backpropagation. The semantic segmentation method proposed in this paper had a higher accuracy than the other methods because it performs backpropagation when the mIOU of the final image does not exceed a certain value.

**Figure 18:**
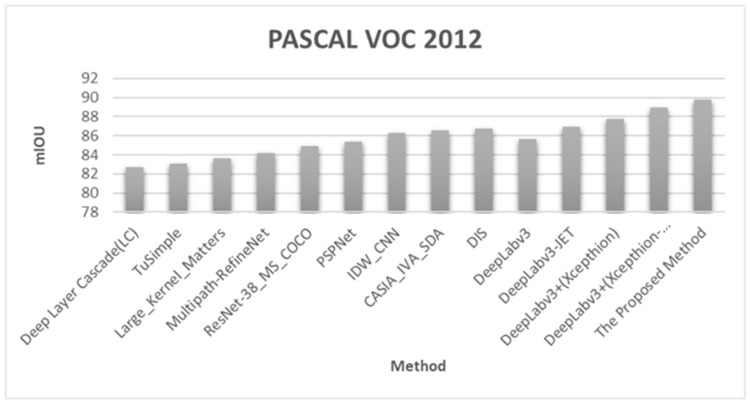
Comparison of the accuracy (mIOU) of the proposed method and methods from the literature on images in the PASCAL VOC 2012 database.

**Figure 19:**
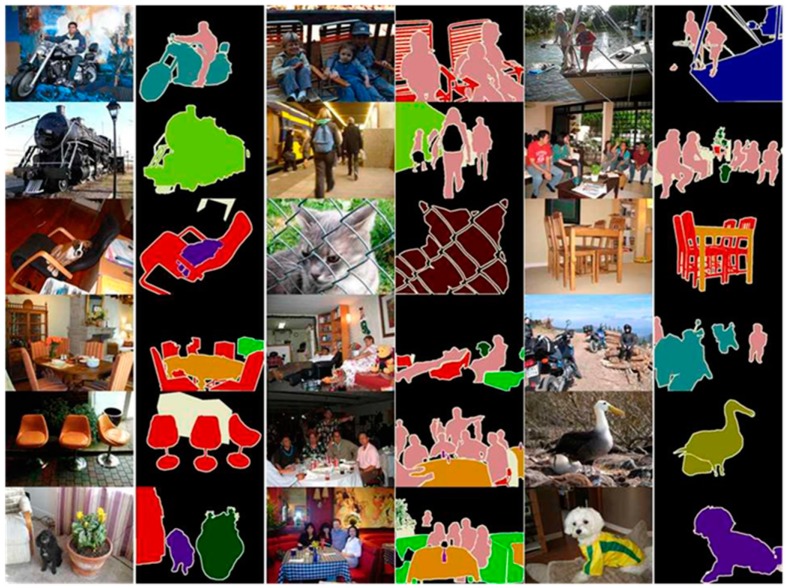
Result of the proposed method for the PASCAL VOC 2012 database photos.

Figure 18 graphically compares the accuracy results for the proposed method and the other methods evaluated by Chen et al. [32] in the same environment for the images of the PASCAL VOC 2012 database. The figure confirms that the proposed method had a higher accuracy than the other methods. Figure 19 shows the experimental results of the proposed semantic segmentation method for the PASCALVOC 2012 database.

In addition, the required time for the proposed semantic segmentation method was measured for the PASCAL VOC 2012 database. Again, we did not compare the required time with other methods because it can vary based on the performance of the hardware on which the program is executed. Table 4 shows the frame rate for the proposed method determined from the required time and the number of images for which semantic segmentation was performed for all images of the database; the result was 64.3 fps. As the frame rate was higher than 60 fps, the proposed method is applicable to the AR field for following human motion.

### 4.3. Custom Result

**Figure 20:**
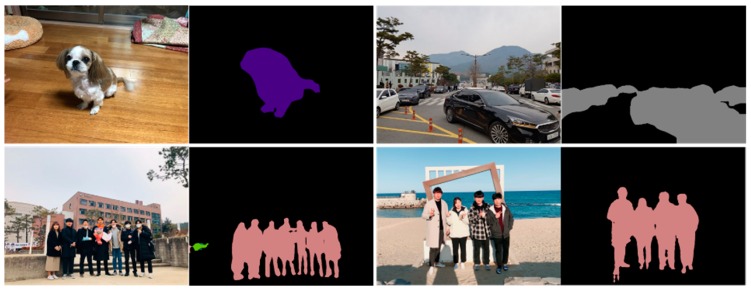
Results of the proposed method for custom photos.

The results of the semantic segmentation by taking pictures of various landscapes and adding noise with a smartphone camera are as follows. Measurement of the mIOU requires accurate background data for the object. However, specifying and comparing the background directly is not reliable, so there is no reason for comparison. Figure 20 is the result of performing a semantic segmentation through the program and determines that the segmentation was adjudged to have been accurately carried out.

## 5. Conclusions

In this paper, we proposed a novel method for semantic segmentation applicable to AR. To evaluate the proposed semantic segmentation method objectively, we used the Cityscapes and PASCAL VOC2012 databases as representative subjects for semantic segmentation. From these databases, the original images and the images for which semantic segmentation had been performed were loaded together. The modified dilated residual network process extracted feature maps through a convolution network appropriate for semantic segmentation by converting the ResNet-101 steps consisting of convolutions. Then, the atrous pyramid pooling module using atrous convolutions in a parallel structure was applied to extract small objects effectively. 

To evaluate the objective reliability of the proposed method, it was compared with methods published in a prior paper using the Cityscapes database and the PASCALVOC2012 database. The results showed that the accuracy and the frame rate were 82.8 mIOU and 61 fps, respectively, for the Cityscapes database, and 89.8 mIOU and 64.3 fps, respectively, for the PASCAL VOC 2012 database. Analysis of the experimental results for the Cityscapes database indicated that for each new technique, the mIOU level increased by less than 0.8 or even decreased. Furthermore, for the experimental results on the PASCAL VOC 2012 database, for each new technique, the mIOU increased by less than 1.2 or decreased. This shows that since the introduction of deep learning techniques [42], it has become very difficult to improve the level of the mIOU, a measure of the accuracy of the semantic segmentation. Therefore, the improved mIOU figures in this paper (Cityscapes: 0.7, PASCAL VOC 2012: 0.8) are considered satisfactory and meaningful. Thus, the proposed method can be applied to the AR field to implement AR natural applications capturing human motion because the frame rate exceeds 60 fps.

To address the accuracy problem, further studies are required to accurately separate the background from complex environments, and on the construction of networks that can be configured effectively for convolution needs. 

## Figures and Tables

**Table 1:** Results of accuracy (mIOU) for the proposed method and methods from the literature on images in the Cityscapes database.

Method	mIOU
ResNet-38 [36]	80.6
PSPNet [29]	81.2
Mapillary [37]	82.0
DeepLabv3 [26]	81.3
DeepLabv3+ [32]	82.1
The Proposed Method	82.8

**Table 2:** Frame rate result for the proposed method with the Cityscapes database images.

Database	Images	Time (s)	Frame Rate (fps)
Cityscapes	1525	25	61

**Table 3:** Results of accuracy (mIOU) for the proposed method and methods from the literature on images in the PASCAL VOC 2012 database.

Method	mIOU
Deep Layer Cascade(LC) [4]	82.7
TuSimple [24]	83.1
Large_Kernel_Matters [22]	83.6
Multipath-RefineNet [38]	84.2
ResNet-38_MS_COCO [36]	84.9
PSPNet [29]	85.4
IDW_CNN [39]	86.3
CASIA_IVA_SDA [40]	86.6
DIS [41]	86.8
DeepLabv3 [26]	85.7
DeepLabv3-JET [26]	86.9
DeepLabv3+(Xception) [32]	87.8
DeepLabv3+(Xception-JET) [32]	89.0
The Proposed Method	89.8

**Table 4:** Frame rate result of the proposed method for the PASCAL VOC 2012 database.

Database	Images	Time (s)	Frame Rate (fps)
PASCAL VOC 2012	17,125	266	64.3
